# *CCL2* single nucleotide polymorphism of rs1024611 implicates prominence of inflammatory cascade by univariate modeling in Indian AMD

**DOI:** 10.1371/journal.pone.0193423

**Published:** 2018-04-17

**Authors:** Neel Kamal Sharma, Kaushal Sharma, Ramandeep Singh, Suresh Kumar Sharma, Akshay Anand

**Affiliations:** 1 Neuroscience Research Lab, Department of Neurology, Post Graduate Institute of Medical Education and Research, Chandigarh, India; 2 Neurobiology Neurodegeneration & Repair Laboratory, National Eye Institute, NIH, Bethesda, United States of America; 3 Centre for Systems Biology and Bioinformatics, Panjab University, Chandigarh, India; 4 Department of Ophthalmology, Post Graduate Institute of Medical Education and Research, Chandigarh, India; 5 Department of Statistics, Panjab University, Chandigarh, India; University of Texas Rio Grande Valley, UNITED STATES

## Abstract

**Background:**

The role of chemotactic protein *CCL2*/MCP-1 has been widely explored in age related macular degeneration (AMD) patients as well as animal models through our previous studies.

**Aim:**

Aim of the study was to examine the association of another variance of *CCL2*, rs1024611 in pathophysiology of AMD.

**Methods:**

This particular SNP has been found to be involved in inflammatory processes in various diseases. Total 171 subjects were recruited in the study with all demographic details by administering a standard questionnaire. SNP analysis was performed with TaqMan assay. Linear univariate and ANCOVA modeling was performed to show the interaction of rs1024611 with another SNP variant of *CCL-2*/*CCR-2* (rs4586 and rs1799865) and impact of individual genotypes on *CCL-2* expression in the context of AMD pathology.

**Results:**

Results showed that both heterozygous (AG, p = 0.01) and homozygous (GG, p = 0.0001) genotypes are associated with AMD pathology. Allele frequency analysis showed that ‘G’ allele is frequent in AMD patients as compared to controls (p = 0.0001). Moreover, AMD patients who smoke were found to be associated with ‘AG’ genotype (p = 0.0145). Although, we did not find any significant interaction between the SNP variants by linear univariate analysis but results show the effect of ‘CT’ genotype on ‘TT’ genotype in rs4586 by considering rs1024611 as covariate.

**Conclusion:**

Based on these results it is imperative that *CCL2* mediated pathology may be associated with AMD.

## 1. Introduction

AMD can be defined with several pathological conditions including drusen formation, macrophages infiltration, apoptosis of retinal cell layers and new blood vessels formation from the choroid. The inflammatory processes have been reported in AMD to result in drusen deposits (dry AMD) which can further provoke the wet AMD pathology. Consequently, these pathological conditions lead to impaired visual function. Chemokine (C-C motif) ligand-2 (*CCL2* or monocyte chemoattractant protein-1) plays an important role in recruitment of monocytes from peripheral blood [1, 2]. The cellular inflammatory processes have been implicated in several degenerative diseases (e.g. multiple sclerosis, Alzheimer disease, arthrosclerosis, rheumatoid arthritis etc.) including cancer.

We wanted to examine whether there is a human link to our previous study in which we showed that *CCL2* produced from mice RPE or choroids facilitates choroidal macrophage recruitment mediated by C5a and IgG as shown in *CCL2*-/- mice study. Therefore, impaired macrophages infiltration would be expected to show AMD features from accumulation of IgG and C5a and further activation of vascular endothelial growth factor (VEGF) [3]. We have also found that ‘TT’ genotype of both *CCL2* (rs4586; p = 0.003) and *CCR2* (rs1799865; p = 0.015) genes is significantly associated with AMD pathology. In case of multivariate analysis the ‘TT’ genotype for both genes i.e. *CCL2* (rs4586) and *CCR2* (rs1799865) were also significantly associated with AMD pathophysiology after adjusting for age (p = 0.005) and gender (p = 0.017) respectively. Moreover, elevated expression levels of *CCL2* and *CCR2* in serum and lymphocytes respectively, in AMD patients, as compared to controls, have also indicated the effect of chemokine ligands and receptors mediating cellular inflammatory processes in AMD pathophysiology [4]. Interestingly, Despriet *et al* did not find any correlation of major alleles in both *CCL2* and *CCR2* haplotypes with AMD patients, however, this study did not include Indian AMD patients. Instead, the minor allele of one haplotype was found to be significant (p = 0.03) with disease phenotypes but there was no effect on mRNA expression profile of these genes in Caucasian population including both Netherlands and USA populations raising the importance of genetic epidemiology in AMD[5].

## 2. Materials and methods

### 2.1 Participants

111 AMD and 60 controls were recruited from Advanced Eye Centre, Post Graduate Institute of Medical Education and Research (PGIMER), Chandigarh, India to conduct the study. Participants were included only after obtaining written consent forms. The ethical clearance of the study was obtained from Institutional Ethical Committee (IEC), PGIMER, Chandigarh, India vide letter number Micro/10/1411.

### 2.2 AMD diagnosis

AMD patients were recruited on the basis of their disease phenotypes observed under fundus angiography (FA) and optical coherence tomography (OCT) by retinal specialists. Various ophthalmic parameters were also screened which included pupils dilation, best corrected visual acuity (BCVA), and opacity of lens by slit lamped microscopy.

### 2.3 Demographic information

Demographic details of the participants were obtained to correlate with the genetic outcome of the study of AMD patients and further compared with controls. The standard questionnaire which includes the set of queries related to food habits, smoking, and their associated co-morbidities (cardiovascular history, hypertension or diabetes etc) were collected (Table 1).

**Table 1:** Demographic characteristics of controls and AMD patients.

Variables	AMD	Controls
Total	111	60
Male	74	39
Female	37	21
Duration of disease¥	24.35 M	—
Dry	28	—
Wet	83	—
Smokers	48	11
Non Smokers	63	43
Vegetarian	59	31
Non Vegetarian	52	23
Comorbidity	81	10
No Comorbidity	28	44
Age	65±7	61±13

Clinical and demographic details of subjects. AMD, age related macular degeneration; M, Months; Age, Age of onset; Values are mean ± SD or (percentage)

¥ Duration of disease is the interval between appearance of first symptom of AMD and collection of sample. AMD subjects were asked to provide all clinical and demographic details at the age of disease-onset.

### 2.4 Inclusion and exclusion criteria

AMD patients were recruited after comprehensive ophthalmological examination by retinal specialist. >5 drusen with size of 125 microns in at least one eye were included as AMD patients. Other pathological features of AMD like leaky blood vessels (by FFA) and degeneration of macular photoreceptors (by OCT) were also included as AMD patients. The participants with less than 5 drusen with size of 60 microns were considered as control subjects. Any pathological conditions resembling AMD phenotypes (e.g. uveitis, retinal dystrophy, vein occlusion, neovascularization due to diabetic retinopathy *etc*) were excluded from the study. The age below 50 years were also excluded from the study.

### 2.5 PBMCs isolation

5ml blood was taken from all participants in EDTA vial and was kept at room temperature for separation of two layers. Supernatant of the samples layered on equal volume of histopaque (SIGMA-ALDRICH, USA) and further centrifuged at 1800rpm for 30minutes. The middle buffy coat were washed with 1X PBS and stored at -80°C for further use.

### 2.6 DNA isolation

Genomic DNA was extracted from PBMCs by commercially available genomic DNA kit (QIAGEN, Germany or INVITROGEN, USA) as per the manufacturer’s instruction. Concentration and purity of genomic DNA were measured by UV spectrophotometer (BeckMan Coulter, USA). The extracted DNA was appropriately coded and stored for further use.

### 2.7 Genotyping assay

Single nucleotide polymorphism (SNP) analysis of *CCL2* rs1024611 was carried out with SNP genotyping TaqMan assay in StepOne real time PCR machine (Applied Biosysystems Inc., Foster city, CA). Reaction set up contained genomic DNA concentration of 20ng and 5μl TaqMan assay (Applied Biosystems). Final volume of the reaction was made up with master mix up to 20μl. Probes were tagged with FAM and VIC dyes to discriminate the allelic changes located at rs1024611 in the SNP assay which posses 5’ nuclease activity. The negative control (without genomic DNA) was also put in reaction setup. The overall protocol for SNP analysis was followed as per the manufacturer’s instruction. The SNP analysis and reaction amplification was done with StepOne V 2.0 software (Applied Biosysystems Inc., Foster city, CA). Fluorescence generated from the SNP discrimination reaction was analyzed by Sequence Detection Software (SDS). The analysis was done between fluorescence amount (Rn value) versus amplification of the products.

### 2.8 Statistical analysis

Genotyping data obtained from SNP analysis was categorized in homozygous and heterozygous variants. The association with SNP changes among various groups was analyzed by Pearson’s Chi square test. Binary logistic regression model was used to get best line fit of distributed genotypes in the population. The correlation with SNP data and strength with disease phenotype (Odd’s ratio or OR) with 95% confidence interval was calculated by logistic regression. All results in SNP correlation with disease pathology were considered significant when analysis p value were less than 0.05.

### 2.9 Linear univariate and ANCOVA analysis

To analyze the impact and/or interaction of rs1024611 (lies in promoter region) on previously published SNP rs4586 (lies in coding region) of *CCL-2* and its receptor rs1799865 (Anand *et al*., 2012) [4], we performed linear univariate modeling. Moreover, ANCOVA analysis was also carried out to test the main effect of rs10246 on other two SNPs and vice versa by assuming any one of them as covariate. We also derived the interaction model further to identify whether presence of one SNP aggravates the AMD pathology. Bonferroni correction analysis for multiple comparisons was done to exclude the false positive outcome of the results.

## 3. Results

### 3.1. Genotype analysis

**Fig 1:**
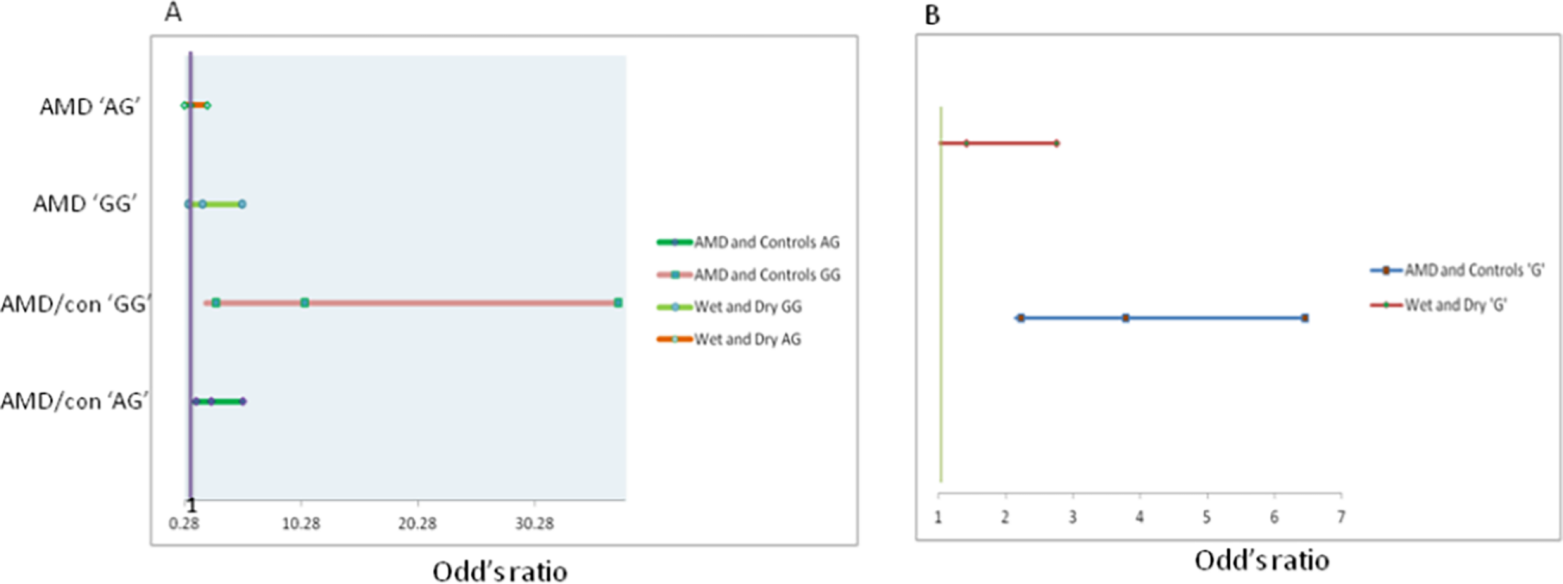
Schematic representation of odd’s ratio (OR) for both genotypes and allele frequencies of rs1024611 locus. (A) Genotypes frequency (B) allele frequency.

The studied population was consisting of 111 AMD patients and 60 controls. The demographic details of the population are given in Table 1. The effect of particular genotype with reference to disease phenotypes has been shown in Table 2. The genotype analysis revealed both heterozygous AG and homozygous GG genotypes have their deleterious effects on AMD pathology as compared with controls (p = 0.01 & p = 0.0001 respectively). On the contrary, any of the genotypes including AA, GA and GG did not demonstrate any association with wet and dry form of AMD. Moreover, the allelic frequency data (Table 3) showed that ‘G’ allele, in comparison to ‘A’ allele in the A/G genotype, has shown significant association with progression of AMD pathology. Similarly, both A and G alleles did not show any effect on both forms of AMD. Moreover, we have also depicted the odd’s ratio (OR) of both allele and genotype frequencies have also been plotted in **Fig 1** for AMD and controls.

**Table 2:** Effect of *CCL2* rs1024611 variants on disease phenotype.

Genotype	Number (frequency)		OR	95%CI	P Value
*CCL2* rs1024611					
	**AMD**	**Controls**			
AA	40 (3.6)	41 (6.83)	Reference		
AG	40 (3.6)	16 (2.67)	2.56	1.24–5.29	0.01
GG	31 (2.8)	3 (0.5)	10.59	2.99–37.43	0.0001
	**Wet AMD**	**Dry AMD**			
AA	29 (34.9)	11 (3.93)	Reference		
AG	33 (39.8)	7 (2.50)	1.78	0.61–5.21	0.28
GG	21 (25.3)	10 (3.57)	0.8	0.28–2.21	0.66

**Table 3:** Allele frequency of *CCL2* in AMD and normal controls.

Allele	Number (frequency)		OR	95%CI	P Value
*CCL2* rs1024611					
	AMD	Controls			
A	120	98	Reference		
G	102	22	3.7864	2.2232 -6.4485	0.0001
	Wet AMD	Dry AMD			
A	91	29	Reference		
G	75	17	1.4059	0.7178–2.753	0.3205

The genotype data was further associated with socio-demographic and co-morbidity variables of the participants (Table 4). The logistic regression analysis demonstrated that heterozygous genotype ‘AG’ has found to be associated with smoking habits and progression of AMD pathology. But both AA and GG genotype haven’t shown any correlation with smoking. None of the genotypes i.e. AA, GA and GG have demonstrated significant correlation with comorbidity. However, GG genotype may have association with comorbidity (p = 0.0625). Food habits of the participants were not found to bear significant association with any of the genotypes.

**Table 4:** Logistic regression of the association of *CCL2* and progression of AMD.

Genotype	Number (frequency)		OR	95%CI	P-value
*CCL2* rs1024611					
	**Non Vegetarian AMD**	**Vegetarian AMD**			
AA	16 (0.31)	24 (0.41)	**Reference**		
AG	23 (0.44)	17 (0.29)	2.0294	0.8329 to 4.9448	0.1193
GG	13 (0.25)	18 (0.30)	1.0833	0.4175 to 2.8109	0.8693
	**Smokers AMD**	**Non Smokers AMD**			
AA	12 (0.25)	28 (0.44)	**Reference**		
AG	23 (0.48)	17 (0.27)	3.1569	1.2554 to 7.9384	0.0145
GG	13 (0.27)	18 (0.29)	1.6852	0.6306 to 4.5035	0.2981
	**AMD with Comorbidity**	**AMD without Comorbidity**			
AA	27 (0.33)	13 (0.46)	**Reference**		
AG	27 (0.33)	11 (0.39)	1.1818	0.4507 to 3.0989	0.7341
GG	27 (0.33)	4 (0.14)	3.2500	0.9394 to 11.2437	0.0627

Using (i) chi-square value (ii) effect size (iii) degrees of freedom used in association (iv) level of significance, and (v) number of observations, the power of the study has been computed. All calculations were made in R software using *pwr*.*chisq*.*test* (w = effect size, N = number of observations, df = degrees of freedom, sig. level = 0.05, power = NULL). By specifying all other parameters, the power has been computed for various associations. In all associations, the power was found to be more than 80%”.

### 3.2. Individual SNPs impact on AMD pathology

**Fig 2:**
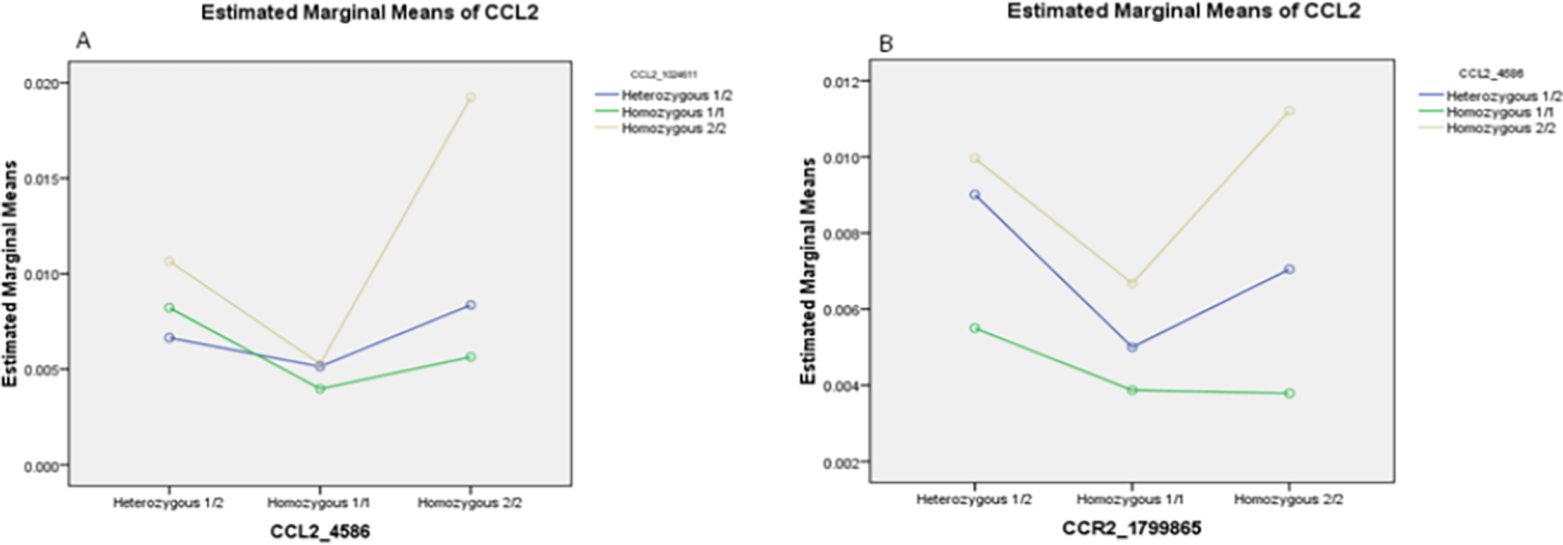
Linear univariate modeling analysis. The interaction shows between (A) rs1024611 and rs4586. Heterozygous 1/2 (AG); homozygous 1/1(AA); and homozygous 2/2(GG) of rs1024611; (B) between rs1799865 and rs4586 with levels of *CCL-2*. Heterozygous 1/2 (CT); homozygous 1/1(CC); and homozygous 2/2(TT) of rs4586.

We have already reported exonic SNP variant of *CCL2* (rs4586) and its receptors (rs1799865) were found to be associated with AMD progression [4]. Promoter SNP variants of *CCL2* (rs1024611) and *CCL2* receptor (rs1799865) interaction was non-significant (F = 1.099; p = 0.359). Moreover, there is no interaction between both *CCL2* SNP variants i.e. promoter and exonic variants (rs1024611 and rs4586) (F = 1.824; p = 0.127) (**Fig 2A**). However, the linear univariate modeling shows that the interaction between both SNPs i.e. rs1799865 and rs4586 is non-significant (F = 0.254; p = 0.907) **(Fig 2B**). Although, Fig 2A shows slight interaction but it is statically non-significant. Therefore, we observed that all 3 SNPs (rs1024611, rs4586 and rs1799865) were impacting the AMD pathology individually.

### 3.3. ANCOVA analysis

Since all the interactions between factors and co-variates were found to be non-significant, therefore, analysis of co-variance was performed with expression levels of *CCL2* as dependent variable and other SNP variants as factors/covariates. For different factors and covariates, the results are presented in Table 5. By considering rs1024611 as covariate and three genotypes of *CCL2* rs4586 (TT = 2, CT = 1 and CC = 0) were compared by taking TT as reference. It was observed that for a fixed reference TT, CT was found to be significantly (p = 0.027) affecting the TT genotype. However, there was no effect on CC genotype (p = 0.356). Similarly, three genotypes of *CCR-2* rs1799865 SNP variant (CC = 2, CT = 1 and TT = 0) were also compared with rs1024611 *CCL2* variants considering as covariate and TT genotype as reference. Remaining both genotypes i.e. CT and CC were not found to be affecting TT genotype (p>0.05). Moreover, in case of *CCL-2* exonic variant as covariate and TT genotype of *CCR-2* variants as reference, analysis showed no significant effect of *CCR-2* rs1799865 genotypes CT and CC on TT genotype (p>0.05). Therefore, impact of genotypes of *CCL-2* (both promoter, the coefficient of rs1024611 is significant, as well as exonic SNP variants) and *CCR-2* genes on reference genotypes may lead to the predictive modeling which may support the experimental evidence of enhanced expression of *CCL-2* in AMD patients as compared to controls, and/or may also modify the binding affinity of *CCL-2* ligand with its receptor (*CCR-2*).

**Table 5:** ANCOVA analysis to determine the affect of genotypes on reference genotype and expression levels by considering one SNP as covariate.

	*CCL-2* levels as dependent variable				
Covariate Factors	**Parameter**	**B**	**Std. Error**	**t-value**	**p-value**
	Intercept	0.007	0.002	4.459	0.000
	*CCL2* rs1024611	0.003	0.001	2.832	0.005
	[*CCL2* rs4586 = CC]	-0.002	0.002	-0.925	0.356
	[*CCL2* rs4586 = CT]	-0.005	0.002	-2.228	0.027
	[*CCL2* rs4586 = TT]	Ref	.	.	
Covariate Factors	Intercept	0.004	0.002	2.410	0.017
	*CCL2* rs1024611	0.004	0.001	3.338	0.001
	[*CCR2* rs1799865 = CC]	0.002	0.002	1.301	0.195
	[*CCR2* rs1799865 = CT]	-0.003	0.002	-1.283	0.201
	[*CCR2* rs1799865 = TT]	Ref	.	.	.
Covariate Factors	Intercept	0.008	0.002	4.725	0.000
	*CCL2* rs4586	0.001	0.001	1.389	0.167
	[*CCR2* rs1799865 = CC]	0.000	0.002	-0.086	0.931
	[*CCR2* rs1799865 = CT]	-0.004	0.002	-1.558	0.121
	[*CCR2* rs1799865 = TT]	Ref	.	.	.

Therefore, we have proposed a univariate model in Eq 1:

$$y_{ij}=\mu +\alpha_{i}+\beta_{j}x_{ij}+e_{\mathrm{ij}}$$ (1)

Where *y*_*ij*_: represent *CCL-2* expression, and

μ: overall general effect

α_i_: effect of i^th^ genotype

x_ij_: covariate

e_ij_: error with mean 0 and variance σ^2^

To rule out the false positive outcome of the results obtained from SNPs interaction and the ANCOVA analysis, we applied the Bonferroni correction for multiple comparisons. It is evident from Table 6 that for *CCL2* rs1024611 the mean difference *CCL-2* levels while comparing genotypes AG versus GG and AA versus GG were significant (p<0.05) whereas AG versus AA were non-significant (p>0.05). However, for *CCL2* rs4586 and *CCR-2* rs1799865 all multiple comparisons were revealed non-significant results (Table 6).

**Table 6:** Multiple comparison using Bonferroni correction analysis to adjust the p values for independent and/or dependent SNPs of rs4586, rs1024611 and rs1799865.

**Benferroni Multiple Comparisons test**						
Dependent Variable: *CCL2* levels						
***CCL2*_4586**		Mean Difference (I-J)	Std. Error	p-value	95% Confidence Interval	
					Lower Bound	Upper Bound
Heterozygous CT	Homozygous CC	.003183	.002289	.383	-.002475	.008841
	Homozygous TT	-.002053	.001702	.485	-.006260	.002153
Homozygous CC	Homozygous TT	-.005236	.002271	.073	-.010850	.000377
**Benferroni Multiple Comparisons test**						
Dependent Variable: *CCL2* levels						
***CCL2*_1024611**		Mean Difference (I-J)	Std. Error	p-value	95% Confidence Interval	
					Lower Bound	Upper Bound
Heterozygous AG	Homozygous AA	.000613	.001738	.940	-.003683	0.004909
	Homozygous GG	-.007647	.002226	**0.003**	-.013149	-0.002144
Homozygous AA	Homozygous GG	-.008260	.002144	**0.001**	-.013561	-0.002958
**Benferroni Multiple Comparisons test**						
Dependent Variable: *CCL2* levels						
***CCR-2*_1799865**		Mean Difference (I-J)	Std. Error	p-value	95% Confidence Interval	
					Lower Bound	Upper Bound
Heterozygous CT	Homozygous CC	0.003228	.002232	.354	-.002289	.008745
	Homozygous TT	-0.000301	.001713	.985	-.004536	.003935
Homozygous CC	Homozygous TT	-0.003529	.002222	.286	-.009023	.001964

## 4. Discussion

The role of chemokine receptors and their ligands in relation to inflammatory processes in AMD is well documented. Most macrophages or microglial cells express the receptors for chemokine ligands and show the chemotactic movements with chemokines gradient at inflammatory site. Both chemokine receptors CX3CR1 and *CCR-2* are expressed on inflammatory macrophages but non-inflammatory macrophages contain only CX3CR1 receptor [6]. Prolonged and persistent existence of macrophages in sub retinal space results in the release of various chemokines and angiogenic factors which consequently stimulate the accumulation of drusen at local inflammatory sites. CX3CR1 variant (M280) have shown the defective migration of macrophages at inflammatory site and found to have enhanced interaction with its ligands in retinal transmembrane [7]. The functional studies have revealed that these cascades of pathological changes in the retinal layers and surrounding microenvironment leads to prominent disease phenotypes i.e. formation of drusen, atrophy of photoreceptors and choroidal neovascularization (CNV), mediated by CX3CR1 signaling [7–10]. Similarly, we have previously investigated the abnormal deposition of C5 and IgG molecules in *CCL2*-/- and *CCR-2*-/- mice due to impaired macrophage recruitment at the site of deposition suggesting the imperative role of macrophages recruitment to clear debris in between retinal layers steered by *CCL2* and *CCR-2* signaling mechanism [3].

*CCL2* genetic studies have not previously shown the association with AMD pathology. Genetic analysis, by considering univariate of both *CCR-2* and *CCL2*, along with TLR4 gene did not reveal any association between studied SNPs and AMD pathology. Even the haplotype analysis in case of *CCR-2* and TLR4 has not shown any correlation with pathology. However, the haplotype analysis of minor allele C35C has demonstrated pathological association (p = 0.03) with AMD pathophysiology in Netherlands and USA populations but mRNA expression did not show significant difference between AMD and control groups [5]. Our investigations have demonstrated that SNP variants of both *CCR-2* (rs1799865) and *CCL2* (rs4586) are associated with AMD pathology. Moreover, the expression of both chemo-attractant proteins was found to be elevated in AMD patients as compared to control groups [4]. Similarly, we have also observed the association of other chemo-attractant proteins including the CCR-3 variants [11] and expression levels of eotaxin-2[12] in AMD patients and further comparison with control groups. Both genes primarily regulate the inflammatory processes by recruiting the eosinophiles and T-lymphocytes mediated mechanisms. Above mentioned studies have suggested the role of cellular processes mediated by chemo-attractant proteins in order to regulate the inflammatory processes in AMD pathology. Interesting finding from Pham et al have demonstrated allelic variance at rs1024611 which leads to allelic expression imbalance (AIE) of *CCL2* which has been reported in various disease phenotypes including atherosclerosis, tuberculosis suggesting that the given allele expression is context dependent which could be influenced with interaction of various proteins [13] that is consistent with our previous findings [4]. Moreover, it has also been explored that *CCL2* expression could also regulate the angiogenic process by affecting VEGF and its associated molecules with the involvement of *Ets*-1 transcription factor [14]. Similarly, our findings with VEGF [15] and its receptor i.e. VEGFR2 have been found to be associated with AMD pathology and the expression levels of both proteins were significantly high in AMD patients as compared to age matched controls [16].

Pathological hallmarks of AMD are similar to age related changes like metabolic changes and apoptosis [17, 18] and enhanced inflammatory responses evident from various age related and inflammatory diseases including Alzheimer’s disease, ischemia, and myocardial infarction [19–21]. In all these studies, the *CCL2* expression was found to be elevated suggesting that AMD pathological phenomenon are induced with inflammatory responses created by various cellular and protein responses. Additionally, we have recently demonstrated the elevated SOD1 levels in AMD as compared to controls, which also show the inflammatory response characterizing AMD [22].

Smoking has also been shown to have impact on various diseases and has been found to be associated with *CCL2* polymorphism and their levels in patients of myocardial infarction [21]. Smoking can also hamper the development of organs in offspring [23, 24]. However, the precise mechanism behind pathological changes induced by smoking in association with genetic markers is being debated. In our finding with *CCL2* it has been shown that heterozygous allele AG is more frequent (p = 0.0145) in smoker AMD patients as compared to non-smoker AMD patients, suggesting a causative role of smoking in possible alteration of genetic allele which may lead to differential expression of *CCL2* protein in the AMD patients [21]. Similarly, logistic regression analysis has also demonstrated correlation of homozygous allele ‘GG’ with co-morbidity (p = .0625) in AMD patients even though it was not significant.

The studies have shown the SNP changes from A to G in enhancer region at -2578 position (rs1024611; A>G) lead to increase expression levels of *CCL2* in various bio-fluids [21, 25, 26] and facilitate the leukocytes recruitment in the tissues [27]. In our earlier observations we have found increased levels of *CCL2* in AMD patients as compared to controls but how rs1024611 influences the *CCL2* expression is still unclear. However, it has been demonstrated by various studies that rs1024611 polymorphism induces the transcriptional activity of *CCL2* gene [28, 29]. ‘G’ allele has found to be induced higher expression of *CCL2* protein in *in vitro* and *in vivo* as compared to ‘A’ allele. Similarly, leukocytes with ‘GG’ genotype as compared to ‘AA’ genotype have also induced increased production of *CCL2* protein. Therefore, these studies suggest the biological impact of the rs1024611 polymorphism in inflammation by recruitment monocytes [21, 26] and its pathological impact on various diseases. We have, however, not analysed the half life and affinity of receptor.

Conclusively, our finding suggests the genetic role of *CCL2* mediated processes in AMD pathology which may lead to infiltration of macrophages and other monocytes thus signifying the importance of inflammatory processes in AMD. It is possible that other environmental changes like smoking may be associated with AMD thus influencing *CCL2* genotype. However, additional studies of *CCL2* genes in South Indian population, which differs in dietary and environmental exposure, based on our current and previous finding with *CCL2*[4], are warranted.
